# Power of rhythms – trains and work along the Baikal-Amur Mainline (BAM) in Siberia

**DOI:** 10.1080/1088937X.2018.1564395

**Published:** 2019-01-08

**Authors:** Vera Kuklina, Olga Povoroznyuk, Gertrude Saxinger

**Affiliations:** a V.B. Sochava Institute of Geography of the Siberian Branch of the Russian Academy of Sciences, Irkutsk, Russia; b Department of Geography, George Washington University, Washington, DC, USA; c Austrian Polar Research Institute (APRI), University of Vienna, Vienna, Austria

**Keywords:** Baikal-Amur Mainline, mobility, railroad infrastructure, travel, work, Siberia

## Abstract

Train rhythms are dictated by regulations as well as the collaboration of human and non-human actants. When a railroad is the prime form of ground transportation and the mono-industry forming force in the cities along the railroad, the rhythms of trains have power over the everyday life of people who rely on them as passengers, workforce and traders. This is the case of the Baikal-Amur Mainline (BAM) in Siberia. The paper tackles the interaction of natural, technical, bureaucratic and economic rhythms and asks where power structures are located. Material and social networks of the involved actants are shaped by constraints and forces: Moscow driven bureaucracy, technological needs and natural conditions as well as the individual or collective needs and aspirations of social beings are entangled within the power structures that are intrinsic to railroad operations. This article is based on ethnographic field work in Siberia along the BAM. In it we argue that the diversity of rhythms introduced by the train company dominates other work and life rhythms that vary across gender, age, class and family status.

## Introduction

The Baikal-Amur Mainline (BAM) is an example of socially powerful infrastructure that serves not only as a ‘city-forming’ but also ‘region-forming’ enterprise, dominating many fields of social, economic and political life of local communities. It includes over 4300 km of railroad, multiple side tracks (with Amur-Yakutsk Mainline being the largest of them) and 200 stations, linking over 60 cities and towns and crossing over 2000 bridges across the territories of six federal subjects of East Siberia and the Russian Far East. While the history of the BAM starts with early construction projects dating back to the nineteenth century and continues with the first tracks laid under the Stalinist regime in the 1950s, the majority of the mainline was built between 1974 and 1984, within the Soviet industrial program of ‘mastering the North’ (Slavin, 1982). The mainline built for resource extraction, became a ‘century project’ employing modern technologies for transformation of the natural environment (Josephson, 1995) and a symbol of Soviet ‘high modernism’ (Scott, 1998) combining the elements of technological and social engineering. The construction process drawing on communist propaganda and ‘the myth of the BAM’ (Ward, 2001) drew mass inflow of labor force, primarily young male workers, from across different parts of the USSR. Many of the migrants settled in the cities and towns they had built to form the majority population with the distinct socio-professional identity of ‘BAM builders’ *(bamovtsy)*. Thus, the railroad became a part of the Soviet project of modernization and internal colonization (Kotkin, 1997), an agent of social change and the backbone of the regional development (Povoroznyuk, 2016).

During Soviet times, railroad workers, consisting primarily of *bamovtsy,* were considered to be part of the military structure at the very core of state power. During the 1990s, when Russia endured a difficult economic transition, railroad workers were part of a rather small group of workers in the country who did not experience withheld wages. The railroad infrastructure also remained heavily subsidized and cargo and passenger trains continued to operate. Currently, the railroad workers, tracks, train stations, locomotives and other related railroad infrastructure are managed by the state-owned monopoly Russian Railroads (RZhD) that was established in 2001. Nowadays Russian railroad work is highly hierarchical, runs to a set schedule, and operates on the Moscow time-zone.

This paper uses BAM infrastructure and its maintenance as an example of the existence of certain configurations of social relations that go well beyond simple movements of people and goods. This also holds true for a railroad’s power to configure rhythms of people involved in its operation and of its passengers.

The regulatory impacts of railroads on social relations has been a focus of research for a long time. Researchers emphasized the synchronization of the rhythms of the railroad with the rhythms of wider social and economic relations (Cottrell, 1939; Gamst, 1980; Kemnitzer, 1977; Lucas, 1971). However, some questions remain that we address in this article: what is the extent of the power of the railroad rhythms? How do natural rhythms such as circadian, seasonality and weather changes inform and at times interfere with the individual rhythms of the work of the railroad? How do the rhythms of railroad maintenance interact with individual and social rhythms like the work of social institutions or the rhythms of households and social networks? How do the rhythms of the railroad affect economic rhythms in the city?

**Figure 1.**
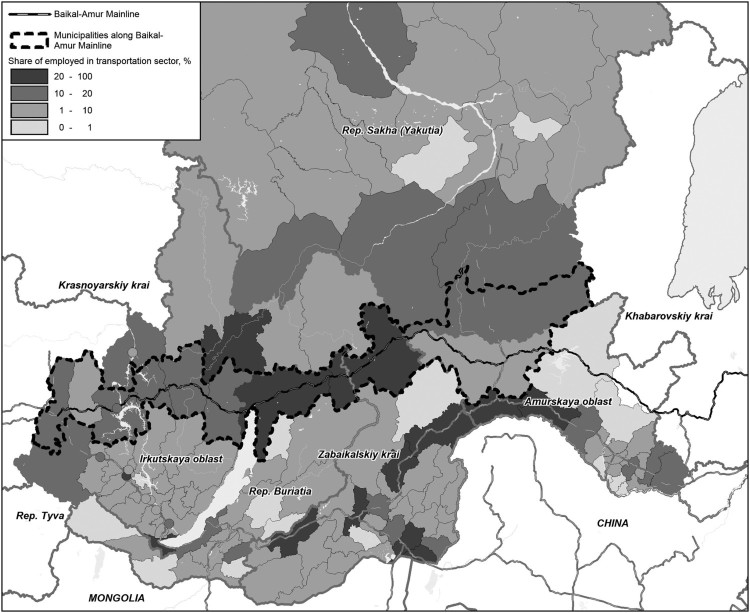
Map of employment in transportation sector along the Baikal-Amur Mainline (made by Viktor Bogdanov).

This paper is based on qualitative field research conducted in the Siberian cities of Ust’-Kut, Tynda and Severobaikal’sk along the BAM in 2016 and 2017. The choice of the cities is based on their importance for the BAM region. While the construction of the BAM railroad started in 1938 and finished in 2003, its main part was built in 1974–1984 under the name of the All-Union Komsomol Construction with millions of young people participating there. Ust’-Kut (population of 42,500 in 2016) was the first city where the active phase of the BAM construction began. It was founded in 1631 by Cossacks and peasants, but the most of growth happened after the construction of the BAM railroad and the opening of Osetrovskiy harbor – the biggest in the USSR – along the Lena river in 1950 into a key transport hub that provides delivery of fuel, materials and non-perishable food to areas with only seasonal transportation access in the northern parts of the Irkutsk region and the Republic of Sakha (Yakutia). It is known as the ‘northern supply’. The main sources of income of the local population are the public sector, transportation, forest industry and the service sector (Saxinger, Petrov, Krasnoshtanova, Kuklina, & Carson, 2016). Severobaikal’sk (population of 23,677 in 2017) and Tynda (population of 33,500 in 2016) are listed as mono-industrial towns with the railroad as the major employer. The former is the center of Severobaikalsk branch of the Eastern-Siberian railroad and the latter – of Tynda branch of Far-Eastern railroad (divisions of the Russian Railroads) while across the whole region employment in transport and communication sector is very pronounced (Figure 1). Other spheres of employment include the public sector and services – shops, cafes and tourist infrastructure (Koriukhina et al., 2012).

In the BAM region the railroad is oriented not as much to serving passengers as to moving cargo: mostly that of natural resources (coal, gas, oil, wood and mineral resources) from Siberia to the Asian markets. With the construction of the ‘East Siberia – Pacific Ocean’ pipeline, the BAM’s significance of oil transport has vanished. However, equipment and workforce are still transported by BAM.

As the BAM railroad was being built, planners strategically placed settlements, locomotive depots and stations with additional tracks, as well as smaller stations for maintenance crew, taking into account the needs of existing settlements but also the specific requirements of the train operation. As a result, the duration of train stops reflects the railroad hierarchy. Bigger stations are required every 6–8 h of the train ride so that engine crews can drive between them in one shift. At these stations, trains stop for a minimum of 30 min in order to change the locomotives, to load water and other supplies to the carriages and dispose of waste. Moreover, Tynda and Severobaikal’sk, as the centers of the railroad management for the respective railroad branches, have also their own locomotive depots and trains. Train crews headquartered and trained there include several hundred train attendants and electrical engineers.

## Methodological context

The field research includes participant observation and a series of 36 in-depth interviews conducted by the lead author with railroad workers, their spouses and representatives of local businesses carried out while traveling along the BAM and in the BAM cities. For each group specific guide for the interviews was created by the lead author and edited by all authors. The questions for the railroad workers included the following: personal biography, main job responsibilities, their schedule and impact on other social and family responsibilities. The spouses of the railroad workers were asked about their biographies, family history, the impact of the spouse work on the railroad on the household’s daily activities. Finally, the interviews with the representatives of local businesses were asked about their experience of interaction with the railroad as an organization, as a carrier and as a material space on a monthly, weekly and daily basis. Based on the answers, the main components of the railroad rhythms were distinguished. Interviews were collected using key respondents and the snowball method. The duration of the interview ranged from 20 to 90 min with the average about 60 min. Materials of the interviews were transcribed and coded.

Our informants were born in this region and have different backgrounds, family histories (Buryats, Evenks, descendants of the peasants who settled in the region in the seventeenth century, first and second generations of the Soviet-time labor migrants who participated in the BAM construction) and family composition (married, single, with and without children). Names and places have been anonymized, and the personal observations presented below reflect the perspective of the lead author.

All three authors were actively involved in the elaboration of the theoretical framework and the analysis of the empirical data laid in the basis of this paper. Field research conducted by the two co-authors in the same region on closely related topics focused on the BAM, provided useful comparative material and facilitated joint discussions on the contents of the paper.

The article focuses on rhythms of long-distance trains to explore the dominating power of infrastructure in multiple ways. It starts with a review of existing approaches to the study of rhythms, their application to railroad studies and the potential of rhythm studies for understanding power relations. In the following parts, we distinguish three main spatial settings where rhythms of the railroad vary in effects on the different actors. First, we observe the effects of the immediate relations of different rhythms of the passengers and train attendants in long-distance trains. It follows by exploration of the ways the rhythms of trains are interrelated with other rhythms of the white-collar and blue-collar railroad workers whose work is conducted at the train stations and along the tracks. The final section explores how the rhythms of the long-distance passenger trains affect the business activities of the BAM cities which include rhythms of entrepreneurs, station kiosks, vendors and bus and taxi drivers. Based on these examples, we argue that the diversity of rhythms introduced by the train operation dominate other work rhythms along the BAM and vary depending on the gender, age, class, family status and travel and work experience of different individuals.

## Theoretical considerations

Our analysis of rhythms is derived from the rhythm-analytical approach proposed by Henry Lefebvre. He noted that ‘everywhere where is interaction between a place, a time, and an expenditure of energy, there is rhythm’ (Lefebvre, 1992/2004, p. 15). Rhythms define the presence and co-presence of certain actors in a certain place at a certain time regulated by physical and biological forces and by social order, such as calendars, schedules and timetables (Zerubavel, 1981). The repetitive, highly scheduled and ordered operation of the railroad lends itself to studying railroad infrastructure through the prism of rhythmanalysis (Lefebvre, 1992/2004).

With the development of Actor-Network Theory, different parts of infrastructure have come to be considered as equal actants of actor-networks (Latour, 2005). It allows to embrace complexities of interwoven ‘natural’, ‘social’ (Krause, 2013) and ‘technological’ processes that form specific rhythms. This also relates to standardization and formalization processes in the field of infrastructures (Lampland & Leigh Star, 2009). The railroad rhythms are the product of orders and regulations as well as incessant collaborative work of interdependent human and non-human actors. In this article, we consider relations between actors in ‘metastable equilibrium’ (Lefebvre, 1992/2004). This means despite the fact that we deal with the repetitive movement of trains, ordered work of the railroaders and cyclical changes of seasons and days and nights, there are no identical moments and there are always certain efforts to keep up with the dominating rhythms. Potential catastrophes and disasters that would break this equilibrium could form another context for research. This equality of attention to different actors does not mean their equal power: some rhythms dominate over others. As a result, they have regulatory power that, according to Edensor, shapes the lives of individuals and groups and makes them conform to dominant routines and timetables or become sidelined as being ‘out of time and step’ (Edensor, 2010, p. 2).

Since almost everything can be studied as a rhythm – from the human body to a city – in this article we focus on identifying the rhythms of the BAM railroad and examining their relations to other rhythms, in particular, the extension of the regulatory power of the state embodied in the railroad and its operation. According to Schivelbusch (1979), the construction and functioning of the railroad were the main reasons for the standardization of time across the world and the shrinking of time and space. As Zerubavel has put it, the railroads serve as effective instruments of state power (Zerubavel, 1981). In the BAM context, standardization of time according to Moscow’s time-zone dominates over ‘task time’ (Ingold, 1995) and ‘private time’ (Zerubavel, 1981). However, the remoteness of a region is illustrative for looking at the limits to the shrinking of time and space found in the ‘friction of distance’ (Hanson & Pratt, 1995), in the forms of technological and physical constraints to increasing the amount of work per amount of time.

The most immediate effect of the dominant rhythms of trains can be found in studies of a railroad travel. There have been several studies in other contexts such as Simonova (2007) who focused on the experience of a passenger traveling along the Trans-Siberian Railroad or Letherby and Reynolds (2005) and Bissel (2016, 2016) observing working passengers on British trains. There are more studies available on short-distance railroad trips, including research on travel time use (Lyons, Jain, Susilo, & Atkins, 2013; Watts, 2008), variety of emotions towards commuting (Edensor, 2010) and configurations of the gender, age and income level of commuters (Stratford, 2014), to name a few. In our paper, we focus only on long-distance trains (meaning journeys of several days) that form specific rhythms of travel.

The regulatory power of the railroad on its workers has been in the focus of attention of scholars for almost a century. In particular, Cottrell emphasized how technological developments increased the power of time over social relations based on the example of the everyday life arrangements of railroad workers (Cottrell, 1939). His observations were further developed by Kemnitzer (1977) distinguishing between ‘clock time’ and ‘switching time’, and Ingold (1995) theorizing on ‘task time’ and ‘clock time’. The ‘task time’ is the relation of one regular activity to another. In the case of locomotive drivers, it means ‘coping with machines’ in order to keep up with the ‘clock time’.

Beyond the immediate effects of the railroad on travel and work, we find it important to examine the power of railroad rhythms in the context of the existing political regime. In particular, our paper engages with the existing literature on post-socialist studies, where differences and rapid changes of temporalities in socialist or neoliberal order are a concern. Verdery’s concept of ‘etatisation of time’ explains how the socialist state would impinge upon and capture the time of its citizens (cf. Verdery, 1996). Ssorin-Chaikov (2017) argues about the inherent failure and continuity of the Soviet modernization where modernity is a temporality corresponding to a particular (in his case socialist) social order. Finally, Kesküla’s case study of Estonian mine workers (2016) shows how differently people relate to new technologies and systematisation of work.

Another important feature we need to take into account in the case of BAM, is the phenomenon of ‘mono-industrial towns’ – cities where most of the economy and employment is defined by a single industry. With the curtailing social and cultural services provided by the state in the Post-Soviet period, the staple industries predefine the whole existence of such cities. According to the studies conducted in this field, the ‘city-forming’ enterprise may dominate not only economic rhythms as one of the results of the lack of alternative economic prospects and path-dependency (Burawoy & Verdery, 1999), but also the everyday life rhythms of the local population (Dimke & Koriukhina, 2012; Koriukhina et al., 2012; Morris, 2016).

A broad range of phenomena examined within the Lefebvre’s rhythmanalytical approach as well as within Actor-Network Theory allows us a freedom of framing our study. We build upon and contribute to the studies of the railroad travel, work and interrelations with other rhythms by examining power relations of both human and non-human actants along and at the railroady. We argue that the predominant rhythms introduced by the state (through train schedules, working hours at the railroad, etc.) intertwine with the everyday life rhythms and shape interactions of involved actants.

## On trains: long-distance traveling passengers and train attendants

The most characteristic features of train travel in Russia are affordability and long distances. At each train station the clocks show Moscow time: as soon as individuals arrive at a train station they relate to a different time and space than from where they have traveled. Regarding the rhythms of long-distance trains, we need to take into account the departure and arrival stations of passengers, duration of travel, frequency and duration of stops and even the rhythm of train movement. The availability of only second- and third-class (*platskartnyy*) carriages is a specificity of the BAM, while on the Trans-Siberian Railroad passengers can choose to travel in first-class carriages. People from all walks of life use the BAM although as already mentioned the overall aim of the BAM construction was to boost cargo transport. Waggons are overseen by conductors who are also in charge for enforcing the passengerś rhythms: for instance, the efforts to regulate biological rhythms can be found in the work of train lighting and restrooms. The lights in the trains are dimmed at 11pm signaling sleep time. From half-an-hour to an hour before and after big settlements there are ‘sanitary zones’ in which the restrooms are locked. The train attendant urges passengers to use restrooms about a half-hour ahead. After smoking was banned on trains, the smokers who follow the rules use the train stops to go outside and smoke.

Meanwhile, the forms and norms of conduct among passengers are regulated informally, based on ‘common knowledge’ that is evident to the lead author as someone who is accustomed to railroad travel and formerly lived in this region. Before travel, passengers spend time preparing for the trip: some purchase tickets in advance, buy food the day before and pack their luggage. Soon after boarding the train, passengers place bags with food near the table. Perishables are for the first day and unperishable food for the rest of the trip. At the stations with at least a 12-minute stop, passengers can buy little pies called *pirozhki*. The conduct of passengers has changed compared to travel experiences a decade ago. For instance, the new policy of the Russian Railroads Company (RZhD) of differentiating the prices for lower and upper berths impacts behavior. While Simonova (2007) described the practice of sharing the table between all passengers in the same compartment, we observed that tables were mostly used by the passengers of the lower berths, while the passengers of the upper berths would wait until those with more expensive tickets had eaten. What has not changed is the need for the passengers on the lower berths to make the bed after each nap or sleeping time to allow a neighbor from the upper berth to have a meal.

Bissel (2016) refers to the railway carriages as a ‘microcosm’ of society. This is especially true for the BAM trains since they are almost the only available kind of transportation and only second-class sleeping and third-class open coach compartments are available on the long-distance trains. During the train journeys in the third-class open coach compartment one can find women traveling with family who make the table look homely with a tablecloth. Mothers with infants create private spaces using sheets as curtains. Teenagers and young passengers spend time with their gadgets as long as the batteries allow. Long-distance commute-workers returning from a work-shift will sometimes be drinking alcohol. Passengers traveling alone use books, magazines and crosswords to avoid communication with neighbors. The use of mobile phones and laptops has not affected social life in the trains as much as in western trains (cf. Bissell, 2010). Their limited use is due not only to the absence of Wi-Fi, but also by the availability of only two sockets for charging batteries in the carriage.

Finally, another specific of Russian trains is the presence of train attendants in each car. They have their own compartment, check tickets of boarding passengers and provide them with bedding, food and drinks. The rhythms of trains frame the train attendant’s responsibilities before, during and after the journey. As the interviewee Lidia (age around 40) puts it:

The train attendant is the host of the carriage. At home it is the host who controls everything. A train attendant does the same. Whatever happens outside, inside and under the carriage, we control the state of the carriage and [are responsible for] the safety of the passengers. There are about 15 carriages [each with train attendants] and one head of the train. If there are real issues, we call the head of the train. And there can be diverse issues: some people die, some people are born, some miss their stop, some bought tickets for the wrong month. There are children or animals in the carriages. Because there are always two train attendants, each working 12 hour shifts, day and night.

The order in which Lidia listed her responsibilities reflects priority of the materiality of the carriage over the social and a focus on routines. She prepares the carriage (boils hot water and cleans) before passengers arrive at the first departure station. Then she checks the tickets of arriving passengers, provides bedding and asks if they need any assistance in making beds or would like to purchase tea, coffee, snacks or souvenirs. During the trip she cleans the restrooms and mops the floor before the main stations, and only if all these tasks are accomplished will she address other issues of the passengers. Her work ends upon the train’s arrival at the final destination. To prepare for the next trip, train attendants collect the bedding and other train amenities (glasses, silverware, blankets) and clean everything well before the train’s arrival to the station. In short, they ensure most of the social control put on passengers by the railway company, such as regulations concerning lights, opening and closure of restrooms, the serving of food and drinks and restricting certain activities.

When the trip takes several days train attendants become familiarized with passengers and may share some local knowledge, such as when the transportation police of the head of the train might check the carriages to make sure that no one is engaged in prohibited activities (e.g. smoking and drinking). The longer the trip, the higher qualified the train attendant must be in order to handle different life issues of passengers. For example, a trip from Severobaikal’sk to Moscow takes four to five days, which means a nine day return trip for train attendants. Lidia describes these long trips as difficult both physically and emotionally. In contrast, another respondent named Boris, who started to work as a train attendant after his health was impaired by seven years of maintenance work, perceived long journeys as less demanding. However, he describes how unusual stable ground under his feet feels for a few days after such a trip.

The train attendant’s long journeys interfere with family rhythms. Lidia is divorced and leaves her daughter with her parents while she is away. Indeed, the psychologist at the station admits that there is a high risk of divorce among women who work as train attendants. The same pattern does not hold for male train attendants. Boris claims to be content knowing his two small children are left with his wife. Another respondent, a station cleaner, who formerly worked as a train attendant, left that job for the one with a smaller salary, because there was no one to stay with her daughter.

Recently, information about a complaints hotline was installed in the carriages. However, there is little possibility to reflect customer satisfaction in wages, except for revoking awards for the attendants. The train attendants working in third-class open coach compartments receive higher wages than the ones working in second-class sleeping compartments because the former is more crowded (50 versus 36 passengers), and more physical work is needed for cleaning and the distribution of food and bedding. According to observations made by B. Rodoman in the 1980s and 2017, both then and now passengers’ requests to make beds would infuriate train attendants (Rodoman, 2017).

As a passenger one experiences the power of the rhythms of trains, train attendants and regulations of biological rhythms. These rhythms interact with physiological needs, norms of conduct with fellow passengers, previous travel experiences and the perception of the journey and service. The non-local passenger associated the repetitive sound of the wheels clanking over the rails and the slow speed of the train with the ‘backwardness’ of the region. As such, it serves as material evidence for a justification of the modernization discourse that the Soviet ideologists produced during the BAM construction and RZhD reproduced in its program of infrastructural development (Povoroznyuk, 2016). The incoming non-local passenger who will work within the program of the BAM technological modernization represents its new ‘wave’ that will again change the rhythms of the region. Meanwhile, the train attendants follow and contribute to the regulatory rhythms of trains mostly as material entities rather than places of social services. The ability to coordinate work and household rhythms remains an important condition for being in this profession.

In this section we learnt how the rhythms of travel by train are formed by a combination of the movement and stops of the train, the frequency and length of train stops, the railroad company efforts to regulate the biological rhythms of the passengers, as well as by the social order formed each journey by a new constellation of passengers. In contrast to Schivelbusch’s (1979) and Bissell’s (2016) observations, long-distance travel on the BAM not only involves the formation of collectives, but verbal conversation and other forms of cohesion between passengers are almost unavoidable. The train attendants also engage with the temporary collectives of passengers, and rather become part of these local collectives than identify themselves with the corporate collectives of the railroad. Their ability to associate with the rhythms of the passengers is a pre-condition for making the long journey more bearable.

## On train stations and tracks: railroad workers

The strict working discipline and occurrence of non-scheduled work define the railroad work rhythms. There are heavy consequences if delays happen so having the railroad workers close to their job has strategic significance. Certain positions require the employee’s residence to be near the place of service (RZhD, 2016), and employees must also be available at different times and different locations to ensure the smooth running of the trains. With the development of mobile communication devices, employees are obligated in terms of time so that they must be within a 10-minute radius of their workplace instead of the former emphasis on being place-bound (e.g. being near a stationary phone). Both white-collar and blue-collar workers can have day and night shifts, and the top-managers are always supposed to be on-call in case an emergency situation arises.

The white-collar railroad workers synchronize their work schedule with local railroad workers, with workers at other stations along the BAM, with the trains and with the company’s head offices in Moscow. Reports and other time-sensitive documents are written according to Moscow time. Sometimes workers operate on Moscow time in regular conversations with each other. The time difference with Moscow affects their routines not only during work, but also their time off:

You do not have private time, always with your cell phone turned on. And at any time you should be available. Q: Why? A: Our system works so that when Moscow works, you work. Their working day has not yet finished and they have questions; then you have to come back to work, explain or finish something. It does not happen very often, but there were cases when I would come back to the office at 11 pm, midnight. It’s good that I live in the proximity. (Olesya, 35)

For the blue-collar workers working outside, such as tracks maintenance workers, rhythms of labor are defined by the work schedule, train schedules, the weather and travel distances. The working day of Ilya (age around 50), a maintenance worker, starts at 8 am with a planning meeting held by the manager who reminds them about health and safety regulations, checks if everyone is sober and informs them about the day’s tasks. At 9 am the workers’ train arrives and takes the crew to the field that can be up to 60 kilometers away. A workers’ train, operating in the morning and evening only, moves railroad workers only. Around noon they have a lunch-break that is scheduled for two hours, but usually lasts only 15 min. Work is mostly conducted in the field and Ilya brings a lunch packed by his wife with him. The work is coordinated with the train schedules; it takes place during the ‘windows’ – time breaks between running trains, which in turn may be affected by delays or accidents.

The workload is regulated by instructions for checking and fixing conditions of the rails along a certain distance of the track, and by environmental conditions. In the winter when the weather is too cold Ilya’s team would start later, after 11 am. When temperatures fall below minus 40°C they secure the rails to prevent damage. In some locations the maintenance workers constructed warming centers with a furnace where they can drink coffee and warm up before going back to work. In summer they may start earlier, around 4 am, before the sun has heated the rails, or they may wait until evening when the heated rails have cooled down. The work is made more difficult by excessive snowfall, thawing permafrost, frost heaves and other technological and natural occurrences on the railroad. The crew members determine the work speed and how often breaks are taken.

When the work is done the crew may be taken by the work car back to their home base. However, crews have a shortage of cars and if there is no car available then the workers must wait, sometimes for hours, for the workers’ train to take them back. Even if the amount of work is minimal, the whole day could be spent in the ‘field.’ The time spent traveling (up to 2.5 h) is partially counted as working hours and partially counted as time off. In order to catch up with his family, Ilya sometimes skips writing reports, which negatively affects his salary and career advancement. In addition, each worker takes turns being on call during nights and weekends in case of an emergency. Despite being home, when on duty Ilya would not plan any trips that take him further than 10 min away from the train station.

Nadezhda (age around 40), the wife of a maintenance worker, found the night shifts and unpredictable duty calls the most difficult part of her husband’s job:

It was very difficult for me to get used to night calls: ‘Where are you going? – To work, I am on duty.’ At night, too, the trains run, and the lights should be lit, and the devices have to work, and everything else. They have a set schedule: working day ends, and then from five [pm] to eight in the morning he is ‘on duty’. During those hours he can be called up at any time, that is, he must be on the phone and in the city.

Zhanna’s (age around 35) work revolves around the cargo trains that stop at the station and varies with the number of wagons on each train. Her shift is 12 h on and 24 h off and is tightly related to the coming and going of the trains. It starts with a cup of tea or coffee shared with work mates in the changing room. She comes in 15 min early for this ritual. They catch up with the latest news at work and about their families and get briefed by the supervisor on any changes to the work schedule. Like most of her colleagues, she smokes. This allows her to have an extra work-break and chat with colleagues from other departments. During extremely cold or hot weather additional pauses are also allowed. The work is only stopped if the temperature outside falls below minus 57°C. The uniform that she has to wear is often a subject of discontent:

In summer we must wear boots, even if it’s plus 40 outside. I am not allowed to take them off even in here [in the office] because it’s my work place. If I take off my shoes and they find out I will have my bonus revoked.

After each train she comes back into the office to type the required report that recently became part of her workday. Together with her colleagues she reflects on the negative changes to their work routine: the work has become more technical with an increased number of instructions that they need to follow and more control measures.

According to Zerubavel (1981), the nights, weekends and holidays are perceived as private time, and the demand to do something in a limited time frame is perceived as an exercise of power relations. The 50-year-old railroad office worker Galina used to work night shifts. With age she noticed worsening health problems, such as confusing day with night, increased tiredness and restlessness, that made her leave that position. Both Zhanna and Galina are married to railroad workers and repeat to each other: ‘*others would not understand.’* If both spouses work at the railroad then they try to alternate their work shifts: if Zhanna has a night shift, her husband takes a day shift and stays at home with the children and vice versa.

Indeed, the necessity to be always within the employer’s reach interferes with the rhythms of households and other institutions in the city. For example, few of the state-owned kindergartens work during the night, at weekends or on holidays. Consequently, due to the irregular work schedule and long distances involved, workers with small children rely heavily on the support of family members, relatives and friends. As time passes, family members get accustomed to the disruptions caused by the railroader’s work schedule. The practice is normalized and serves as a common ground for discussions with colleagues.

In this section we highlighted several examples of the rhythms of railroad work that are affected by the RZhD regulations (like schedules of trains) and how these rhythms interfere with human and non-human rhythms. The strict discipline required of workers, which resembles life in the military, forms the context of railroad work. For white-collar workers we find more need to coordinate their work with higher-up offices. For blue-collar workers extreme weather conditions and the long distances involved interfere more with the scheduled rhythms of work. Meanwhile, social rhythms including household responsibilities and socializing needs are either embedded into the work routine (drinking tea and chatting at work, both spouses working on the railroad) or sacrificed for one’s career. Household activities are left to other family members or interfere with the work and subsequently career advancement.

## On the train station and beyond: entrepreneurs

The effects of the railroad vary among different professions. While there is a variety of commercial activity connected to the use of trains, we will focus on the most visible forms: on smaller businesses, including entrepreneurs, using trains for the delivery of goods, on kiosks, street vendors and taxi drivers.

For goods transportation entrepreneurs usually use postal-baggage cars attached to passenger trains. The passenger trains are faster although more expensive than the cargo trains. According to interviews with entrepreneurs, transportation by passenger train adds about 25–30% to the cost of goods in the stores while the goods transported by cargo train have only a 5% mark-up. The higher costs of using passenger trains make it difficult for small companies to compete with big firms. Particularly, two bigger retail companies have increased their presence in the local markets, gradually displacing smaller enterprises. The stations where passenger trains do not stop make the delivery of perishable products more complicated, and at the stations with a 2–5 min stop the amount of cargo has to be small enough to allow unloading on time. As a result, the longer the train stops the more opportunities there are for business development.

Despite the modest number of passenger trains – only three to five long-distance passenger trains a day operate in each direction – the timing and duration of their stops correlates with the location and opening hours of kiosks and cafes at and around the stations. In Ust’-Kut the regular passenger trains usually stop during the night, and not many passengers take a walk around and shop, so there are only two kiosks at the station. In Severobaikal’sk the trains stop during the day. The train station has five kiosks and a clothes market and several cafeterias are located within a 10-minute walk. Although the rhythms of their work need more observation, one can easily notice the dependence of kiosks’ working hours on the train schedules and frequencies. They are busier in summer time when there are more passenger trains. The traditional image of Trans-Siberian train stations – vendors offering food on the platform – has vanished with growing security measures and RZhD regulations. Nevertheless, at Uoyan station hawkers still sell smoked fish, beer or soda to passengers when there are no police or railroad representatives around.

The transportation police – part of the RZhD organization – are permanently present at the main stations while the regular police have less staff and make less effort to control the territories around the train stations. As a result, one can find individuals offering fish and souvenirs adjacent to the stations on land belonging to the local municipalities.

Igor (age around 40), a street vendor selling smoked fish (mostly omul) in a summer time and frozen – in the winter, explains that in order to sell goods at the train station he would have to rent a place from the RZhD on a permanent basis. Currently, he sets up stall on land adjacent to the train station and sells his products only when passenger trains arrive (only 2–3 trains every other day) and leaves as soon as the train departs (in less than an hour). Due to his stall being located on municipal territory, he pays a small income tax to the local municipality. His main customers are passengers and train attendants of the passing trains. Other people can find lower prices at the local market located beyond the reach of the train passengers.

The local buses and taxis coordinate their schedules with the arriving and departing passenger trains. In big Russian cities passengers can order a taxi online. In 2017, the BAM cities did not yet have a taxi app. Possibly this is because taxi services are already cheap and taxis will be waiting at the station. As in many other cities due to the limited number of jobs (cf. Koriukhina et al., 2012; Morris, 2016), the BAM cities have numerous taxi service companies and informal taxi drivers (*bombilas*).

Andrey, 50 years old, is one of those *bombilas* who specializes in greeting passengers from the trains. His work rhythms are adapted to the passenger train schedule. He arrives at the train station almost an hour before the train’s arrival in order to occupy a better parking space. He approaches and offers his taxi services to passengers who have alighted on the railroad platform. His fare is quite a lot higher than normal for the city, so he looks for those arriving for the first time or who are unfamiliar with local fares. During the rest of the day he is busy finding short-term work in other places or laboring at his summer house (*dacha).* His earnings hardly cover his own expenses.

Stepan (age 30), another taxi driver, works for a taxi company during his time-off from his more income-generating job as a rotational shift worker in the extractive industries (see Saxinger, 2017) where his schedule is one month on, one month off. Mostly he drives passengers who have already been in the city before or who live there and call to order a taxi. Another group of frequent passengers are the railroad workers ‘on duty’ who may be called upon at any minute. Since the taxi fare is very low, such trips are sometimes cheaper than using personal cars. Stepan calls it ‘harvest time’ when the railroad workers receive their salaries: ‘*everybody knows when payday is on the railroad: local stores try to bring in more food and goods by those dates.*’

In this section we highlighted the most evident effects of the railroad rhythms on local businesses. The contrast between Ust’-Kut with better roads and a more diverse local economy and Severobaikal’sk with a limited choice of transportation and jobs helps us to see how the latter is more dependent on train schedules, regulations of cargo transportation and other internal RZhD policies. As there are fewer transport options, the railroad in Severobaikal’sk has more regulatory power on the rhythms of other institutions. This is reflected in the monthly circulation of money, in the deliveries of goods, in the daily schedule of buses and in the clustering of markets, kiosks and vendors near the train station. Beyond the physical parameters, such as the availability of side tracks and the location of train stations, the work rhythms of entrepreneurs are affected by the train schedule, length of the passenger train stops and frequency of trains. The owners of kiosks and cafes at the trains stations and street vendors adjust their working hours to the schedules of the trains, and opening times depend on the timing and length of the passenger train stops. The fishermen and producers of local souvenirs, supplying goods to the street vendors, get indirectly affected by the railroad rhythms.

The location of vendors on or beyond the territory of the train station shows a marked contrast between the RZhD’s efforts at orderliness and the city’s limited capacity to diversify its economy. Taxi drivers coordinate their work with the arrival and departure time of the passenger trains. Such work does not generate prosperity, but, in contrast to the highly regulated railroad work regime, it offers more ‘time flexibility’ (Morris, 2016). Thus, the rhythms of trains are adapted to by the local entrepreneurs and coordinated with many other rhythms that form their economic strategies and larger everyday rhythms. However, the case of entrepreneurs also demonstrates the limits of local adaptation to the dominant rhythms. The smaller businesses gradually retreat under the pressure of the larger firms privileged by the railroad company.

## Conclusion

In this article, we have shown how the focus on rhythms can help to examine the effects of the trainline infrastructure on the everyday lives of individuals who travel by or work on the railroad and in related fields. While previous research has emphasized the dependence of locomotive drivers and other workers on the rhythms of railroads, our case study covers a much wider variety of actors. Our ethnography of different groups involved with the railroad illustrated the role of rhythms in the organization of power relations. Economic, administrative, technological and natural rhythms of the railroad interact and impact on the workforce, passengers and traders in railroad cities. A specific feature is the domination of the Moscow time zone according to which the railroad – in this case the Baikal-Amur Mainline (BAM) – operates.

The bureaucracy of the Russian Railroads Company (RZhD) headquartered in Moscow impacts white-collar workers in remote railroad cities through a set of regulations. At the same time, the rhythms of nature – a harsh cold climate and darkness in winter or hot temperatures during the summer – interfere with blue-collar workers undertaking their daily work along the railroad. Passengers are drawn into the microcosm of a train by temporal regulations concerning the use of toilets and bedtime hours and the train space defining their interaction with fellow passengers. Traders – be it shop keepers, petty traders or freight handlers operating at the stations – need to adjust their business activities to train schedules. Such impact stretches far beyond the coming and going of trains to include increasing competition between small and bigger businesses, the economic niches they occupy, the purchasing tactics of individuals and local mobilities in general.

Independence from the power of the railroad is achieved by some local residents at a cost: the passengers and businesses have to use other more expensive modes of transportation, and the former railroad workers retreating to the field of entrepreneurship find new forms of domination of the railroad over their everyday routines. As the case of vendors illustrates, even fishermen leading a traditional way of life do not exist completely autonomously from the railroad infrastructure.

While the complex organization of a railroad is a top-down process imposed by the railroad company, people still make sense out of it and create new rhythms altering the organization of their daily life and work. The inhabitants of railroad cities organize child care, daily meals, partnerships or family life in patterns dependent on their gender, age, class and family, but, most importantly, by the dominating railroad rhythms. Bureaucracy and technological regulations imposed by the Moscow-based administration, as well as natural conditions and biologically defined human needs, shape networks of actors involved in power relations that are intrinsic to daily routines and business activities on and along the railroad.
